# Immunohistochemical Estimates of Angiogenesis, Proliferative Activity, p53 Expression, and Multiple Drug Resistance Have No Prognostic Impact in Osteosarcoma: A Comparative Clinicopathological Investigation

**DOI:** 10.1155/2008/874075

**Published:** 2009-02-25

**Authors:** Flemming Brandt Sorensen, Kenneth Jensen, Michael Vaeth, Henrik Hager, Anette Mariane Daa Funder, Akmal Safwat, Johnny Keller, Mariann Christensen

**Affiliations:** ^1^University Institute of Pathology, Aarhus University Hospital, Aarhus Sygehus, THG, 8000 Aarhus, Denmark; ^2^Department of Oncology, Aarhus University Hospital, Aarhus Sygehus, NBG, 8000 Aarhus, Denmark; ^3^Department of Biostatistics, University of Aarhus, 8000 Aarhus, Denmark; ^4^Department of Orthopaedic Surgery, Aarhus University Hospital, Aarhus Sygehus, NBG, 8000 Aarhus, Denmark

## Abstract

*Purpose*. To investigate angiogenesis, multiple drug resistance (MDR) and proliferative activity as prognostic variables in patients suffering from osteosarcoma. *Methods*. Histologic biopsies from 117 patients treated in the period from 1972 through 1999 were immunohistologically investigated regarding angiogenesis (CD34), proliferative activity (MIB-1), and the expression of p53 and MDR (P-glycoprotein (Pgp); clones JSB-1, C494, and MRK16). Quantitative and semiquantitative scores of immunoreactive cells were analyzed statistically along with retrospectively obtained clinicopathologic variables. *Results*. Chemotherapy reduced the rate of amputations (*P* = .00002). The Pgp was overexpressed (score ≥2) in 48% of the primary, diagnostic biopsies, and high Pgp correlated with high Pgp in postsurgical specimens (*P* = .003). In contrast, no such associations were disclosed for estimates of angiogenesis (*P* = .64) and p53 (*P* > .32), whereas the MIB-1 index was reduced in the post-chemotherapy specimens (*P* = .02). The overall, disease-specific survival was 47%, increasing to 54% in patients receiving pre-operative chemotherapy. Statistical analyses showed prognostic impact exclusively by patient age and type of osteosarcoma. *Discussion*. The studied series of patients documented already prior to the chemotherapy era, a rather excellent survival and estimates of angiogenesis, proliferation, p53, and Pgp expressions, did not demonstrate sufficient power to serve as predictors of treatment response or survival.

## 1. INTRODUCTION

The treatment of patients with osteosarcomas has during the last two decades changed from a solely surgical approach to a highly complex multimodal therapy with preoperative chemotherapy as the dominating innovation. Prior to the chemotherapy era, the studies addressing survival of patients with osteosarcoma have, among others, identified morphological type of tumor, site of tumor, tumor stage and size, and duration of symptoms as prognostic variables of relevance [1, 2]. Investigations including large patient populations treated after the introduction of preoperative chemotherapy protocols have evidenced a shift toward histological response to chemotherapy as the most informative prognostic parameter [3–5], a conclusive finding also corroborated by meta-analysis [6] and large-scale studies [7].

Although the treatment outcome for patients with osteosarcoma has improved significantly regarding survival after the introduction of preoperative chemotherapy, it is still difficult to optimize the therapy offered to the individual patient in that the most important prognostic variable cannot be evaluated precisely until after surgical intervention. The extent of tumor necrosis can be guided by various image scans, but first after completion of preoperative chemotherapy, objective measures in the histopathological examination of the surgical specimen are able to estimate the true extent of chemotherapy-induced tumor necrosis [8, 9].

Attempting to get around this obstacle, researchers have focused their investigations on the characteristics of the primary, diagnostic biopsy from patients with osteosarcomas. Features like proliferation rate [10], *P53* gene alteration [11], and especially, multiple drug resistance (MDR) [12] have been examined. Findings regarding prediction of chemotherapeutically induced tumor necrosis have, however, been rather contradicting, and thus difficult to implement in a routine diagnostic setting of osteosarcoma. Moreover, recent results from a randomized phase III trial of the *European Osteosarcoma Intergroup* suggest that intensified chemotherapy may improve histologic response but not survival in osteosarcoma patients [13]. This questions the idea of using the extent of chemotherapeutically induced osteosarcoma necrosis as a surrogate measure of clinical outcome.

The development of new microvessels in tumors, the so-called neoangiogenesis, has for some years been investigated in a number of different types of neoplasms, and has indeed been shown to be of prognostic significance in some. Estimating the level of angiogenesis in a malignant neoplasm is attractive for several reasons, and may reflect the capacity for a malignant tumor to metastasize, and from the therapeutic point of view, for developing antiangiogenetic drugs that may lead to starvation and ultimately death of the tumor in question. The angiogenetic level may also be of interest in planning systemic chemotherapy, as it is the case for patients with osteosarcomas, and it has accordingly also been investigated in such neoplasms [14–18].

In this retrospective study, we have investigated the prognostic value of angiogenesis, proliferation rate, alterations in p53 and MDR/P-glycoprotein (Pgp) expressions in osteosarcomas from patients treated at the Sarcoma Center, Aarhus University Hospital, Denmark from 1972 through 1999, using an immunohistochemical approach. The study thus includes patients treated both before and after the introduction of preoperative chemotherapy, and provides an update of the treatment results in patients with osteosarcoma at Aarhus University Hospital.

## 2. PATIENTS

The cohort of patients studied was retrospectively retrieved from the files of the University Institute of Pathology, Aarhus University Hospital, Denmark in the period from 1972 through 1999. This database was combined with the clinical database at the Department of Oncology, Aarhus University Hospital, Denmark, and a total of 134 consecutively admitted patients treated for primary, nonmetastatic osteosarcoma of the extremities and pelvis were identified. Most of the patients had been referred to the Sarcoma Center of Aarhus University Hospital. Retrieval of clinical follow-up information and/or histological material was unsuccessful in 17 cases, leaving a total of 117 patients for inclusion into the investigation with at least 5 years of follow-up after primary treatment.

The clinicopathological data of the studied patients are summarized in Table 1. Prior to 1984, the treatment of osteosarcoma at the Sarcoma Center, Aarhus University Hospital was limited to surgery, but after 1984, preoperative chemotherapy was offered to all patients, followed by surgical treatment which has increasingly focused on limb-salvage resections. In the early chemotherapy era, the MAP-regimen (mitomycin, adriamycin, and cisplatin) [19] was used, based on *Mayo Clinic* experience. From 1991, the chemotherapy was changed to the *European Osteosarcoma Intergroup Study* approach, based on doxorubicin and cisplatin [13]. A few patients received chemotherapy and/or radiation therapy alone or in combination (Table 1), mostly as an adjuvant postoperative therapy at recurrent disease, in that only very few patients refused surgery at primary treatment.

The clinical data were retrieved from the medical records. Patients were seen at regular intervals as outpatients at the Department of Oncology, Aarhus University Hospital, or at admission to this Department or the Department of Orthopaedic Surgery in the case of recurrence. Thus, any type of recurrence was added to the clinical records and to the clinical database, and in the case of death, the cause of death was established from autopsy records or from the death certificates. The investigation was approved by the Ethical Committee of Aarhus County (Project no. 1999/4601) and by the Danish Data Registration Authorities (Danish Data Protection Agency, Project no. 2000-41-0174).

## 3. MATERIAL AND METHODS

Primary, diagnostic tumor biopsies, formalin fixed, and paraffin embedded, from all 117 patients and tumor specimens from the 112 patients, who underwent surgical treatment, were available for investigation. One representative tissue block was selected from each case by screening all histological sections. In this way, 117 tissue blocks from primary, diagnostic biopsies, and 46 tissue blocks from surgical resection specimens of patients receiving preoperative chemotherapy were selected for the investigation. When possible, the tissue blocks were selected from the soft tissue extensions of the osteosarcomas, in that this tissue had underwent none or only a short period of decalcification in either nitric acid or EDTA.

The surgical specimens from patients treated with preoperative chemotherapy had been processed according to international guidelines of estimating the extent of chemotherapy-induced tumor necrosis [8, 9]. Briefly, the resected osteosarcoma was sawed along the largest diameter of the tumor, and a slice of the surgical specimen was subsequently decalcified and embedded in paraffin blocks in toto. All histological sections cut from each tissue block were examined and a semiquantitative estimate of overall tumor cell necrosis in percent was reported. The tissue blocks used for further studies were carefully selected to ensure vital tumor tissue, but in 6 cases with 100% tumor necrosis, no further investigations were performed.

### 3.1. Immunohistochemistry

Histologically, 4 *μ*m thick sections were cut from each tissue block, placed on coated slides, and deparaffinized in xylene followed by ethanol. Endogenous peroxidase was blocked in methanol with H_2_O_2_ (v/v 35%). Several experiments were carried out to unmask epitopes, including various enzymatic treatments and buffer baths in micro-oven, but the fragile, decalcified tissue sections mostly detached from the histologic slides. The best results were obtained by immersing the histologic slides in TEG buffer (pH 9.0) at 60°C in a heating cupboard for 72 hours, followed by sensibilization in TRIS buffer (pH 7.6) and normal horse serum (DAKO, Glostrup, Denmark).

Incubation with primary antibodies for detecting proliferation (clone MIB-1, DAKO, Glostrup, Denmark), p53 (clone DO-7, DAKO, Glostrup, Denmark), and CD34 (clone QBend10, Immunotech, Quebec, Canada), diluted 1 : 200, 1 : 200, and 1 : 100, respectively, in antibody diluent (DAKO, Glostrup, Denmark) was performed overnight (18 hours), followed by rinsing in TRIS buffer. Secondary biotinylated antibody (PK-6200, Vector) was added for 1 hour, followed by TRIS buffer added 0.5% blocking reagent (DuPont TSA Kit). To intensify the staining, the histologic sections were treated with tyramide (in TRIS buffer added H_2_O_2_) and rinsed in TRIS buffer. Avidin-biotin complex served as the tertiary antibody (PK-6200, Vector), with incubation for 1 hour followed by rinsing in TRIS buffer. Diaminobenzidine (DAB) was used as chromogen, and finally the histologic sections were counter stained in Mayer's hematoxyline and cover slipped in Aquatex. The tyramide signal amplification (TSA) showed excellent results for staining with MIB-1 and p53, whereas it had no improvement on the staining for CD34, which was overall of rather poor quality with only 95 assessable biopsies out of a total of 163.

The immunohistological staining for MDR (Pgp) was carried out by employing three different monoclonal antibodies, clones JSB-1, C494 (Zymed Laboratories Inc., Calif, USA), and MRK16 (Alexis Biochemical, Lausen, Switzerland), diluted 1 : 20, 1 : 500, and 1 : 400, respectively. Visualization was performed by the Envision technique (DAKO, Glostrup, Denmark). Preliminary trials showed rather different staining results of individual biopsies with the 3 different antibodies, and accordingly it was decided to perform the staining procedure of all cases with a primary antibody cocktail containing all 3 clones, which has also been recommended by an international consensus report regarding detection of Pgp [20]. All immunohistochemical procedures were followed by negative controls, omitting the primary antibody. For MIB-1, p53, and CD34, the tumor sections had an inborn positive control, and sections from normal human kidney were used as positive control for the Pgp staining.

There were no differences in the quality or scorability of the immunohistochemical stains, when comparing osteosarcomas included early or late in the nearly 30-year uptake period of the study.

### 3.2. Scoring of MIB-1 and p53

**Figure 1:**
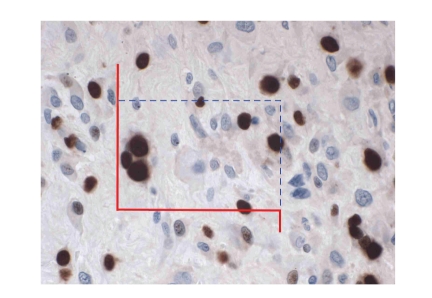
Field of vision showing an osteosarcoma stained immunohistochemically by MIB-1 to demonstrate cells in proliferation cycle (brown nuclear stain). A counting frame has been superimposed onto the histologic section for estimation of the rate and index of neoplastic proliferation. Using an *unbiased* two-dimensional counting rule, nuclei in focus inside the frame or on the hatched, blue edges are counted, as long as they do not intersect with the fully drawn, red exclusion edges of the frame or their extensions. In this example, 6 nuclei in cycle (brown) and 10 “resting” nuclei (blue) are counted (original magnification: 400X).

The immunoreactions for both MIB-1 and p53 are nuclear, but a few cases showed in addition a weak, cytoplasmic reaction, which did not, however, interfere with the morphometric quantification of the sections, in that the tyramide signal amplification technique highly intensified the nuclear staining. The quantification of the MIB-1 and p53 immunoreactivity was carried out, using the same morphometric approach, applying the computer-assisted CAST-grid system (Olympus, Denmark). At low magnification, the histological sections were projected onto a computer screen, using a video camera. The computer software enables drawing around the tumor area (the so-called meander), and within this area, the computer generates fields of vision at high magnification (1425X), with the first field generated at random, and the subsequent fields distributed systematically within the meander area. A counting frame was superimposed on the histological image, and using an unbiased, two-dimensional counting rule [21], the positive and negative nuclear profiles were counted (Figure 1). The ratio of positive to negative nuclear profiles was calculated in percent, and, moreover, the density index of positive nuclear profiles pr. mm^2^ tumor area was calculated. On the average, 50 fields of vision (range: 18–103) were investigated in each tumor (corresponding to a mean tumor area of about 80 mm^2^), with a mean of 140 counted nuclear profiles per case (range: 5–542). The sampling fraction within the studied tumor sections ranged between 0.7% and 6.7%.

### 3.3. Estimation of angiogenesis

**Figure 2:**
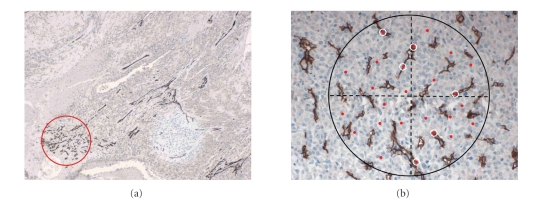
(a) Low-power view of a histologic section from an osteosarcoma, showing a microvascular hot spot in the lower left corner (original magnification: 40X). (b) Histologic section with the Chalkley graticule superimposed. The graticule, equipped with 25 stochastically spaced points, is rotated until the highest number of these 25 points coincides with immunohistochemically stained microvascular profiles in the tumor tissue. The mean of three hot spot counts is reported as the angiogenetic Chalkley number (immunohistochemical stain by CD34 to highlight endothelial cells and vascular profiles; original magnification: 200X).

The histological sections were scanned at low magnification to localize the three so-called *hot spots* with increased microvascularity (Figure 2(a)). In each of these hot spots, the neoangiogenesis within the tumor tissue was quantified using the Chalkley technique [22], as recommended earlier [23]. This method uses a graticule, mounted in the ocular of the microscope, with 25 randomly distributed points. At high magnification (250X), the ocular was rotated until the highest number of these 25 points superimposed on microvascular profiles in the tumor tissue (Figure 2(b)). The mean of the three hot spot counts was reported as the angiogenetic Chalkley number. In cases with no obvious hot spots, the mean of three randomly selected fields of vision within the tumor area represented the Chalkley number. As mentioned above, the angiogenesis could not be scored in a large number of cases because of a false negative immunostain. This is probably caused by an inherent defect in the studied tissue sections, may be due to acid exposure during decalcification at the primary tissue processing, in that a lot of microvascular profiles could be demonstrated morphologically.

### 3.4. Scoring of MDR/Pgp

**Figure 3:**
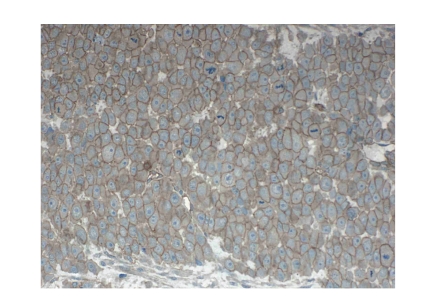
Histologic section of an osteosarcoma stained by a “cocktail” of antibodies against P-glycoprotein (MDR). In this case, the stain shows intense membranous accentuation with a sparse cytoplasmic reaction (MDR score = 3; original magnification 200X).

The scoring of Pgp immunostaining was carried out on a semiquantitative scale, in accordance with earlier studies performed at the *Rizzoli Clinic,* Italy [12]: score 0 = complete absence of immunoreactive tumor cells; 1 = scattered, weakly immunoreactive tumor cells in less than 10% of the histological specimen; 2 = diffuse, weakly immunoreactive tumor cells in more than 10% of the tumor area; 3 = diffuse, intense immunopositivity in more than 10% of the tumor cells. A Pgp score ≥2 was considered to represent resistance to chemotherapy [12, 17]. The heterogeneity of the Pgp immunostain within one tumor section, and among different tumor sections, was only moderate, with just a few cases showing marked variability in staining intensity, and the scoring was based on the highest degree of immunoreactivity within the examined histological slides. Mostly, the Pgp positive cells showed a diffuse cytoplasmic immunoreactivity, but in some cases, an enhanced membrane staining was visualized (Figure 3), as was reinforced immunoreactivity of the Golgi region. Complete absence of Pgp-staining was only recorded in 13 specimens, and in one specimen the Pgp-reactivity could not be estimated because of recurrent detachment of the histological section from the slide during processing.

## 4. STATISTICS

Associations between categorical variables were assessed by *χ* ^2^-test or Fisher's exact test. Student's *t-*test was used for comparison of continuous variables between groups of patients. A rang sum test was used if the variation in the data was poorly described by a normal distribution. A paired *t-*test or McNemar's test was used for pre versus post comparisons. Associations between continuous variables were assessed by Pearson's correlation coefficient and Spearman's correlation coefficient for nonnormal data.

Survival times and time to relapse were described by Kaplan-Meier plots and the prognostic evaluation of the variables was performed by log rank tests; the continuous variables were dichotomized at the median. Patients were followed until death from disease, or until the latest clinical control at the closure of the study. A 5% level of significance was used for all statistical tests.

## 5. RESULTS

### 5.1. Clinicopathologic data

The obtained clinicopathological data are summarized in Table 1. Seventy one (61%) and 46 patients (39%) date from the time before and after the introduction of preoperative chemotherapy, respectively.

On the average, the diagnostic delay was estimated to nearly half a year, but with a wide range, with 11 patients having a delay for more than one year. This may explain the fact that the vast majority of osteosarcomas evidenced soft tissue extension at diagnosis, with only 19% of the patients presenting with a tumor limited by cortical bone. Nineteen tumors (16%) represented other types of osteosarcomas, than the conventional high-grade neoplasm. This group of patients showed a heterogeneous range of morphological types, including both small cell and telangiectatic osteosarcomas, and osteosarcomas of lower grade malignancy.

Five patients did not receive surgical treatment, whereas 26 patients underwent limb-salvage surgery in combination with preoperative chemotherapy. The majority of the patients had amputation which represents a reflection of secularity related to the retrospective design with patients treated within a period of 27 years. Although histological evaluation of the surgical specimens showed intralesional resection in 4 cases, the clinical response to treatment was judged incomplete (persistent disease) in 10 patients. However, the latter group includes also 5 patients, who did not receive primary surgical treatment, whereas one patient was found to have metastatic disease shortly after surgical treatment.

In Table 2, the relationship between the type of surgical treatment, documented by the histological examination of the surgical specimen, and the administration chemotherapy has been listed. There is a significant shift from amputations to surgical treatment by wide resections with the introduction of preoperative chemotherapy (*P* = .00002). Only 35% of the patients receiving this treatment modality had, however, excellent histologic response (>90% tumor necrosis).

### 5.2. Quantitative variables

Results regarding angiogenesis, proliferation, and p53 expression are summarized in Table 3. Separate analysis of these variables in the group of nonconventional osteosarcomas showed that these parameters, on the average, were not statistically different from the results harvested from the investigated conventional osteosarcomas (.06 < *P* < .20). However, in this group, also including low-grade osteosarcomas, the angiogenesis, proliferation rate and index showed a tendency toward lower values (5.94, 43.2%, and 1565, resp.), whereas the p53 expression and p53 index showed a tendency toward higher values (74.17 and 3277, resp.). The elevated p53 scores were specifically caused by extremely high values of this parameter in a few round cell osteosarcomas included in this limited group of patients.

Data on angiogenesis in both pretreatment and posttreatment histological specimens were available in 25 patients, and no statistically significant difference in the degree of microvascularity was disclosed in the two series of biopsies (*P* = .64). Analyses among the other quantitative variables investigated showed mostly no statistically significant associations (*P* > .10), apart from various associations between estimates of proliferation and the p53 expression in the studied osteosarcomas. Thus, estimates of the pre-chemotherapy p53 expression were inversely associated with both pre- and post-chemotherapy estimates of proliferation (*P* < .04). On the other hand, estimates of the post-chemotherapy p53 expression were positive associated with the same estimates of tumor cell proliferation (*P* < .02). These associations, however, also are reflections of the decreased proliferation in post-chemotherapy osteosarcomas (Table 4), which was statistically significant regarding estimates of proliferation index (*P* = .02). No associations were detected between the quantitative variables and the largest tumor diameter (*P* > .12) or clinical parameters (*P* > .10).

### 5.3. MDR/P-glycoprotein

A total of 60 primary, diagnostic biopsies (52%) showed a Pgp score less than 2, and 56 biopsies (48%) expressed a score higher than 2, whereas one biopsy could not be evaluated. In the group of nonconventional osteosarcomas, the cases considered to be chemotherapy sensitive constituted 63% of the cases. The Pgp expression showed no correlation with the investigated quantitative and semiquantitative parameters (angiogenesis, *P* > .15; proliferation rate, *P* > .54; proliferation index, *P* > .51; p53 expression, *P* > .13; p53 index, *P* > .22).

Six out of 46 osteosarcomas were associated with 100%, chemotherapeutically induced tumor necrosis, and in 3 cases the post-chemotherapy specimens could not be immunohistochemically evaluated. In the former 6 osteosarcomas, the corresponding Pgp expression in the pre-chemotherapy, diagnostic biopsies showed a score of 1 (i.e., chemotherapy sensitivity) in 4 cases, score 2 (chemotherapy resistance) in one case, and for the remaining case the pretreatment biopsy could not be evaluated for technical reasons.

In the 37 osteosarcomas, where post-chemotherapy biopsies were available, there was no statistically significant difference in Pgp expression in the pre- and post-chemotherapy biopsies, respectively (*P* = .10), although the MDR-score changed from resistance to chemotherapy sensitive status in 4 cases (Table 5). High values of the MDR-score in the pre-chemotherapy biopsies were positively correlated with high values of the same parameter in the paired, post-chemotherapy tumor specimens (*P* = .003). Moreover, the expression of Pgp in the primary, diagnostic biopsies was not predictive of the degree of chemotherapy-induced tumor cell necrosis (*P* = .53; Table 6).

### 5.4. Prognostic evaluation

The overall disease-specific survival in the entire series of patient, from inclusion to closure of the study period, was 50%, with the group of patients receiving chemotherapy showing a survival of 54%, and in the cohort of patients prior to the era of chemotherapy the overall survival was 47%. Regarding recurrence-free survival, the same figures were 48%, 53%, and 45%, respectively. The group of patients with nonconventional osteosarcomas showed no statistical differences regarding the quantitative variables investigated, when compared to the whole series of patients investigated (.06 < *P* < .20), and accordingly, patients with all morphological tumor types were aggregated into one prognostic analysis. Moreover, separate statistical analysis of patients treated before or after the introduction of chemotherapy showed no individual differences for the investigated quantitative variables. Accordingly, the results of the prognostic evaluation are presented for the whole series of patients.

**Figure 4:**
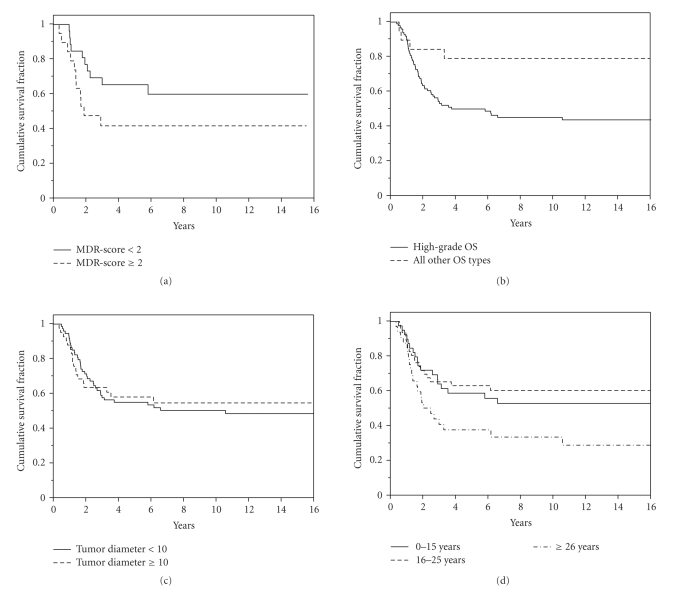
Kaplan-Meier plots of survival in patients with osteosarcoma regarding (a) multiple drug resistance score (*P* = .12; log rank test), (b) histological type of osteosarcoma (*P* = .02), (c) tumor diameter (*P* = .11), (d) and patient age at diagnosis (*P* = .03).

Patient sex, tumor site and extent, diagnostic delay, and type of surgical treatment were without prognostic impact, whereas patient age and histological type of osteosarcoma showed prognostic value regarding survival (Table 7; Figures 4(b), 4(d)). Thus, high-grade osteosarcomas and age > 25 years were associated with poorer survival. Large tumor diameter may carry a tendency to shorter survival, however, nonsignificant (Table 7; Figure 4(c)). Interestingly, neither the administration of preoperative chemotherapy nor the histological response to chemotherapy (i.e., the extent of tumor necrosis) provided any prognostic impact (Table 7).

The data obtained regarding angiogenesis, proliferation rate and index, p53 expression and index, and Pgp expression (Table 8 and Figure 4(a)) were without any prognostic value, neither regarding patient survival (*P* > .12) nor recurrence of osteosarcoma (*P* > .11).

## 6. DISCUSSION

Among the traditional clinicopathological variables, only the type of osteosarcoma and patient age showed prognostic impact regarding survival which is in agreement with other studies of patients with conventional high-grade sarcomas [1, 2, 5], but in contrast to earlier investigations. The size [2, 4, 5, 7] of the tumor, patient sex [7], and diagnostic delay [1, 2, 7] were nonsignificant regarding prognostic power. In this study, older age, surgical treatment by amputation, no surgical treatment and conventional high-grade osteosarcoma were indicative of an unfavorable prognosis. This may be a reflection of the characteristics of the database in that the patients have been collected over a period of about 30 years. Moreover, the group of nonconventional osteosarcomas also contained tumors of lower-grade malignancy.

On the other hand, it is surprising that neither preoperative chemotherapy nor histologic response to that treatment showed prognostic impact. These results disagree with most large studies of patients treated by preoperative chemotherapy for osteosarcoma [3, 4, 6, 7], except for a recent randomized phase III study by the *European Osteosarcoma Intergroup* [19]. The latter multicenter investigation could not document any relationship between the degree of histologic response to chemotherapy and patient outcome regarding survival [19]. The results of our study may reflect matters of secularity related to the database investigated in that the rarity of osteosarcoma necessitate collecting patients for study over decades,a time frame within which minor or even major changes in treatment may occur. An overall, disease-specific patient survival of 47% before the introduction of preoperative chemotherapy is rather high, when compared to earlier studies [1, 2], and considering the very long follow-up presented in the present study. With such a high survival rate before the introduction of preoperative chemotherapy, the true value of the treatment may not penetrate from the statistical analysis. One has, however, to consider the retrospective design of the present study, which also implies possible bias due to patient selection and data confounding, especially related to treatment variables. Nevertheless, the benefit of chemotherapy was reflected by the shift from domination of amputation to preferential limb-sparing and wide surgical resection before and after the introduction of chemotherapy, respectively.

The fraction of proliferating tumor cells in the investigated series of osteosarcomas ranged from 5% through 90%. This represents a larger range than that obtained in some studies [10, 24–26], but corresponds to the figures reported by German investigators [27, 28]. We used the TSA technique to improve the often rather weak immunohistological staining for MIB-1, which may explain our higher range of osteosarcoma cells in proliferation cycle. The discrepancies may, however, also be caused by a large number of confounding parameters, associated with the different immunohistological techniques and antigen retrieval methods, and dealing with retrospective, paraffin-embedded archival tissue, that is, the standardization of fixation methods is questionable. Furthermore, it has been shown that the Ki-67/MIB-1 antibody may stain noncycling cells that overexpress p53 [29], which may indeed be the case in osteosarcomas [26].

There was no significant reduction in the proliferation rate in post-chemotherapy specimens, but a statistically significant decrease in the proliferation index was demonstrated. This is, however, just a reflection of the used arithmetic, in that the proliferation index is related to the area of tissue investigated, and not the number of MIB-1 negative cells, and the area without tumor cells increases when chemotherapy makes its effect.

It has been suggested that MIB-1 could be used as an adjuvant variable in the morphological classification of primary bone tumors because low-grade osteosarcomas have been shown to have a lower proliferation rate than conventional high-grade sarcomas [24], and low-grade osteosarcomas have a higher MIB-1 rate than fibrous dysplasia [30]. With the problems associated with immunohistochemical techniques, mentioned above, and our finding of nonsignificant, lower values of MIB-1 rate in nonconventional osteosarcomas, we would advise against employing MIB-1 estimates for guidance in classification of osteosarcomas.

An increased rate of proliferation in metastases, when compared with the MIB-1 rate in the corresponding primary osteosarcomas, has been published [31], and thus adds evidence to the suggested role of MIB-1 rate to act as an indicator of biological behavior of osteosarcomas [25]. Prognostic impact of the proliferation rate has been reported in patients with both skeletal [32, 33] and extraskeletal [34] osteosarcomas, in contrast to our findings, which, however, are corroborated by other investigators [10, 26].

Most studies addressing the immunohistochemical expression of p53 in osteosarcomas have used a semiquantitative scoring approach of the staining results [35–40], often with a 10% or 20% threshold for scoring a tumor as p53 positive. Using this approach, the immunoreactivity for p53 has been used in attempts to classify bone tumors [39, 41]. We have used a strictly quantitative, morphometric technique, in accordance with an earlier editorial on this issue [42], which explains a frequency of 100% of p53 positivity in our series of osteosarcomas. The staining pattern, and thus the difference in p53 reactivity, was highly different among individual tumors, showing a range from 5% through 100% p53-positive tumor cells. Our reservations in interpreting the immunohistochemical results for MIB-1 are, however, also valid for p53. In addition, we experienced a rather dramatic heterogeneity in p53 staining within the same slide from one tumor, and will accordingly recommend a random-systematic sampling approach in immunohistochemical studies of immunohistochemical cellular p53-expression.

In accordance with our results, immunohistochemical investigations of p53 in patients with osteosarcomas have not demonstrated prognostic impact [26, 31, 43]. Changed p53 protein expression may not convey mutation in the *P53* gene [36], and accordingly, genetic studies of *P53* gene alteration may be of value. However, a few studies based on molecular technology in evaluating the *P53* gene in osteosarcomas have not been able to document prognostic value in osteosarcomas [11, 44, 45]. Nevertheless, it may, from the theoretical point of view, still be valuable to study various suppressor genes like *P53* and their protein expressions, in that osteosarcomas are known to occur with higher frequency in persons with impaired or defect suppressor genes like in the case of inherited retinoblastoma [46, 47]. Indeed, a recent study has shown prognostic impact of the combined information regarding protein expression of markers of apoptotic cell death, such as p53, bax, and bcl-2, as compared to missing prognostic value displayed by each of these molecules individually [48]. Also, the combination of p53 with information regarding Pgp has been documented to contain stronger prognostic information than the two variables separately [49]. Thus, the true prognostic value of p53/ *P53* on either the protein or gene expression level, respectively, awaits further clarification.

The rate of Pgp positive osteosarcomas in the present investigation was comparable to findings in other studies [50–52], but on the average, often a little higher than reported by other investigators [12, 53, 54]. Again, these discrepancies can be caused by differing techniques employed, and may not reflect differences related per se to the patient populations studied. Missing correlations between Pgp expression and the level of proliferation and p53 expression are in agreement with earlier findings [55].

Although showing a tight correlation, the Pgp expression was not different in primary, diagnostic biopsies and post-chemotherapy specimens in the present series of patients with osteosarcomas, and the Pgp level in the primary biopsy could not predict the extent of chemotherapy-induced necrosis. The latter findings have been reported by other investigators [12, 51, 52], and a meta-analysis including 631 patients has confirmed the missing predictive power of Pgp expression with regard to the extent of chemotherapy-induced tumor necrosis [56]. Our methodological approach cannot, however, provide reliable answers to whether Pgp expression is an accurate measure to predict chemotherapy resistance, in that tumor cell necrosis in itself may not be an accurate measure for this resistance.

The incidence of Pgp overexpression has been shown to increase after chemotherapy [52], but our findings cannot be compared reliably with this study, in that the two investigations use quite different scoring schemes for evaluating the MDR-status. Although the incidence of Pgp expression has been shown to be increased in metastatic as compared to the corresponding primary osteosarcomas [53, 54], this does probably not imply the development of a more aggressive phenotype [57], and experimental data suggest that MDR is not upregulated in the course of tumor progression [58].

The prognostic impact of MDR/Pgp expression in osteosarcomas is highly debatable. Some studies have shown Pgp overexpression in primary, diagnostics biopsies to be predictive of poor prognosis [12, 50, 53, 59], which may be of relevance in planning the chemotherapy [60]. Other authors have not been able to detect prognostic value of the Pgp expression, as determined by immunohistochemistry [40, 61]. Some of the confusion may be related to differences in documenting and scoring immunohistochemical Pgp expression. We have employed the international recommendation of using cocktails of two or more vendor-standardized anti-Pgp antibody reagents that recognize different epitopes [20]. This may improve the reliability of the immunohistochemical, overall detection of Pgp, but the maybe unique clinical or prognostic impact of the individual antibody clones used remains undetected by our approach.

The immunohistochemical overexpression of Pgp may not, however, reflect true genetic amplification of the MDR-gene [62], and quantitative RT-PCR analysis of osteosarcomas from 123 patients was unable to demonstrate any relationship between the genetic MDR expression and prognosis [63]. So far it seems that most investigations, using immunohistochemical study design, favor prognostic significance of Pgp overexpression, which also is the conclusion of the meta-analyses mentioned above [56].

Investigations of angiogenesis in human osteosarcomas are rather sparse, but some studies have shown prognostic impact of estimates of angiogenesis, either directly [15–17] or indirectly [64, 65], whereas others have not been able to prove any prognostic value by angioenesis [18]. Some research based on animal models suggests, however, a role for angiogenesis in the evaluation of progression of osteosarcoma [66], or in the treatment of such tumors with antiangiogenetic drugs [67, 68]. We could not demonstrate any prognostic impact by angiogenesis, and were unable to find any significant differences in the degree of vascularity in pre- and post-chemotherapy tumor specimens. Moreover, there seems to be no obvious relationship between the grade of angiogenesis and the expression of Pgp in individual osteosarcomas, pointing to the conclusion that angiogenesis is not associated with the accessibility of chemotherapeutics to the vascular bed of the tumor cells. Only a nonsignificant tendency to lower estimates of angiogenesis in nonconventional osteosarcomas was revealed.

We have used the Chalkley technique [22], as recommended by Vermeulen et al. [23], whereas other studies [14–18, 64] on human osteosarcomas have used the microvessel density score for estimating the grade of vascularity within tumors investigated. Although estimates of angiogenesis obtained by these two different approaches may be correlated, a direct comparison is not possible. The sampling approach for both techniques is based on estimation within preselected *hot spots.* Dealing with the highly heterogeneous vascular morphology in osteosarcomas, this sampling method seems rational. Optimal sampling schemes, like random systematic sampling, as used for scoring of proliferative activity and p53-expression, seemed in our hands unsuited for angiogenesis estimation. Necrosis, condroblastic, and bony-differentiated areas within the often small diagnostic biopsies from osteosarcomas, as related to the focality of neovascularisation (see Figure 2(a)), made such stereological approach for estimating architectural structures like vessels futile, in contrast to the suitability of this methodology in quantifying cellular events like p53-expression and proliferation.

Furthermore, in our investigation, we have experienced great difficulties in the immunohistological staining by antibodies to CD34, reducing the number of cases available for statistical analysis regarding patient survival and recurrence of osteosarcoma. In our preliminary investigations, we also tested out the usability of CD31 for immunohistochemical detection of neoangiogenesis. However, CD31 showed an immense, nonspecific background staining in the decalcified tissue sections, making it impossible to obtain reliably quantitative estimates of angiogenesis.

Also, the end points for various analyses regarding prognostic impact of angiogenesis are varying, in that some studies focus on survival and recurrences, whereas others primarily are addressing the response to preoperative chemotherapy, another point disabling bona fide comparisons between various investigations. Moreover, the number of cases in the studies, published so far, is rather sparse. Adding together all these problems associated with estimation of neovascularity in osteosarcomas, we believe it is too early to dismiss angiogenesis as a possible parameter for prognostic evaluation, and for purposes of planning treatment in the event antiangiogenetic drugs are introduced in treatment trials of patients with osteosarcomas.

In searching for new morphologic and molecular predictors of prognosis and treatment outcome, the oncopathologist is faced with the fight against tumor heterogeneity [69]. Indeed, this battle seems insurmountable if one considers the sparseness of the primary, diagnostic biopsy from the mostly bulky osteosarcomas. The intratumoral heterogeneity may be attributed to intrinsic, tumor cell-specific characteristics or caused by the environment of the tumor growth. Moreover, the tissue sampling within the tumors and the technical approach used makes heterogeneity one of the most difficult obstacles to manage in both a scientific and clinical contexts, even in the case of monomorphic, small cell malignancies like, for example, Ewing sarcoma [70]. The diagnostic biopsy from an osteosarcoma will always represent a keyhole of the neoplastic reality. Dealing with cellular events like p53 expression and proliferative activity, systematic random sampling can combat some of the problems related to heterogeneity. Angiogenesis is more difficult in this regard, and a semiquantitative approach seems most feasible for quantifying such architectural aspects. Monitoring the expression of MDR/Pgp is also highly sensitive to tumor heterogeneity, but one molecular study, aimed at this particular issue, has revealed very little variation of intratumoral MDR expression [71]. The overall impact of all these problems suggests humbleness in interpreting, and in taking clinical consequence of studies of prognostic variables in all kinds of human neoplasms.

## 7. CONCLUSION

This retrospective, immunohistochemical study of patients treated for osteosarcoma does not disclose prognostic impact of quantitative estimates regarding angiogenesis, tumor cell proliferation, p53 status, or P-glycoprotein expression. Likewise, the use of preoperative chemotherapy and the extent of induced tumor cell necrosis were without prognostic value. Although the study has a retrospective design which may inflict data confounding and bias, these findings may be associated to the fact that the overall survival was around 50% even before the introduction of preoperative chemotherapy. Conflicting evidence in the literature regarding the true, prognostic impact of the dogmatized, predictive value of the histopathologic response to preoperative chemotherapy in patients suffering from osteosarcoma may ask for intensified research regarding the pretreatment, predictive value of, for example, patient-specific qualities like MDR status.

## Figures and Tables

**Table 1:** Clinicopathological data of 117 patients with primary osteosarcoma.

	N
*Sex*	
* *Women	* *41
* *Men	* *76
*Age*	
* *Mean (years)	* *23.8
* *SD (years)	* *16.6
* *Range (years)	* *4–74
*Tumor site*	
* *Humerus and radius	* *12
* *Pelvis	* *10
* *Femur	* *66
* *Tibia	* *21
* *Fibula	* *6
* *Tarsal bones	* *2
*Extent of tumor*	
* *Intraosseous	* *6
* *+ Cortical breakthrough	* *16
* *+ Soft tissue extension	* *95
*Type of osteosarcoma*	
* *Classical high grade	* *98
* *Other types	* *19
*Largest tumor diameter* ^a^	
* *Mean (cm)	* *10.1
* *SD (cm)	* *3.9
* *Range (cm)	* *3–20
*Diagnostic delay* ^b^	
* *Mean (months)	* *5.3
* *SD (months)	* *3.9
* *Range (months)	* *1–36
*Type of surgical treatment*	
* *None	* *5
* *Tumor resection	* *26
* *Amputation	* *86
*Histological radicality of surgery* ^c^	
* *Intralesional resection	* *4
* *Marginal resection	* *5
* *Wide resection(radical)	* *49
* *Amputation (radical)	* *54
*Chemotherapy* ^d^	
* *+ Preoperative chemotherapy	* *46
* * − Preoperative chemotherapy	* *63
* *+ Other kind of chemotherapy	* *8
*Radiation therapy* ^e^	
* *+ Radiation therapy	* *7
* * − Radiation therapy	* *110
*Clinical response to primary treatment*	
* *Complete clinical response	* *107
* *Persistent disease	* *10
*Histological response to preoperative chemotherapy*	
* *100% tumor necrosis	* *6
* * >90% tumor necrosis	* *11
* *50–90% tumor necrosis	* *14
* *<50% tumor necrosis	* *10
* *No necrosis	* *5
*Recurrent disease* ^f^	
* *No recurrence	* *51
* *+ Recurrence	* *56
* *Persistent disease	* *10
*Survival status* ^g^	
* *Survivors	* *58
* *Death caused by OS	* *58
* *Death from unrelated disease	* *1

^a^Tumer diameter not known for 3 patients. ^b^Diagnostic delay was estimated from the anamnestic informations given in the clinical records and was unknown for 19 patients. ^c^Five patients did not receive surgical treatment, but were by own wish treated by either chemotherapy and/or radiation therapy alone/combined or refused any treatment. ^d^One patient received chemotherapy as the only treatment modality and did not wish the subsequent surgery, whereas 7 other patients received chemotherapy as adjuvant to other treatment. ^e^Two patients received radiation therapy as the only treatment. ^f^Recurrence status at the closure of the study. ^g^Survival status at the closure of the study.

**Table 2:** Histological radicality of surgery in relation to preoperative chemotherapy course^a^. *P* *value* ^b^ = .00002.

Type of histological radicality of surgical specimen:	Preoperative chemotherapy not given	Preoperative chemotherapy given	∑
Intralesional or marginal resection	5	4	9
Wide resection	19	30	49
Amputation	43	11	54

∑	67	45	112

^a^Five patients did not receive surgical treatment, but were by own wish treated by either chemotherapy and/or radiation therapy alone/combined or refused any treatment. ^b^ *χ* ^2^-test.

**Table 3:** Angiogenesis, tumor cell proliferation, and nuclear p53 expression in primary, diagnostic biopsies from osteosarcomas.

	N	Mean	SD	Range
Angiogenesis^a^	95	6.79	2.50	2.00–14.00
Proliferation rate (%)	117	50.6	18.8	5.1–90.2
Proliferation index (mm^−2^)	117	1990	1164	14–8388
p53 expression (%)	117	68.0	24.4	5.5–100.0
p53 index (mm^−2^)	117	2615	1437	15–10556

^a^It was technically impossible to obtain evaluable immunohistochemical stains for CD34 in 12 cases.

**Table 4:** The relationships between estimates of tumor cell proliferation and nuclear p53 expression in 46 osteosarcomas, before and after preoperative chemotherapy.

	Pre-chemotherapy		Post-chemotherapy		
	N	Mean	N	Mean	*P* * value* ^a^
Proliferation rate (%)	46	54.48	39^b^	42.8	.86
Proliferation index (mm^−2^)^c^	46	2230	39^b^	1327	**.02**
p53 expression (%)	46	61.0	39^b^	64.8	.85
p53 index (mm^−2^)^c^	46	2573	39^b^	2190	.32

^a^Student's unpaired *t*-test. ^b^One case could not be evaluated, whereas 6 cases were associated with 100% tumor necrosis. ^c^Index reflects the number of positive nuclear profiles pr. mm^2^ tissue.

**Table 5:** Multiple drug resistance (Pgp expression) in 37 osteosarcomas in pre- and post-chemotherapy biopsies, respectively^a^. *P* *value* ^c^ = .10.

Pre-chemotherapy MDR-score^b^	Post-chemotherapy MDR-score^b^		∑
	<2	≥2	
<2	20	1	21
≥2	5	11	16

∑	25	12	37

^a^Three cases could not be evaluated in the post-chemotherapy specimens, and 6 cases were associated with 100% tumor necrosis. ^b^MDR-score ≥2 indicates resistance to chemotherapy. ^c^McNemar's test.

**Table 6:** Relationships between multiple drug resistance (Pgp expression) in pre-chemotherapy biopsies of 46 osteosarcomas and histological response (necrosis) in surgical specimens^a^. *P* *value* ^b^ = .53.

Pre-chemotherapy MDR-score^a^	Tumor necrosis		∑
	<90%	≥90%	
≥2	12	7	19
<2	16	11	27

∑	28	18	46

^a^MDR-score ≥2 indicates resistance to chemotherapy. ^b^Fisher's exact test.

**Table 7:** Analyses of prognostic impact of clinicopathological, categorical variables, grouped as shown in Table 1.

	Survival^a^	Recurrence^b^
Number of patients	116^c^	107^d^
	*P*-*values* ^e^	*P*-*values* ^e^

Sex	.16	.33
Age^f^	**.03**	.79
Extent of tumor	.91	.90
Type of osteosarcoma	**.02**	.06
Largest tumor diameter^f^	.11	.36
Diagnostic delay^f^	.30	.13
Type of surgical treatment	.16	**.05**
Preoperative chemotherapy	.78	.31
Histological response to chemotherapy^g^	.33	.48

^a^Survival analysis based on disease-specific mortality. ^b^All kinds of recurrences, but patients with persistent, progressive disease after primary therapy have been deleted from the analyses. ^c^One patient, who did not receive preoperative chemotherapy, died from unrelated disease, and has been censored from the statistical analysis. ^d^Six and 4 patients, who did not or did receive preoperative chemotherapy, respectively, had persistent, progressive disease after primary treatment, and are excluded from the statistical analysis. ^e^ *P*-values as reported from log-rank tests. ^f^Analysis based on a 3-group comparison, divided on the tertiles. ^g^Analysis based on a comparison between patients (showing ≥90% versus <90% tumor necrosis, resp.).

**Table 8:** Analyses of prognostic impact of quantitative and semiquantitative immunohistological estimates, as obtained from the pretreatment biopsies (number of patients analyzed shown in brackets).

	Survival^a^		Recurrence^b^	
	no pre-chemotherapy	+pre-chemotherapy	no pre-chemotherapy	+pre-chemotherapy
	*P*-*values* ^c^		*P*-*values* ^c^	

Angiogenesis^d^	.90	.92	.23	.76
	(65)	(30)	(65)	(35)
Proliferation rate (%)	.56	.52	.82	.54
	(70)	(46)	(65)	(42)
Proliferation index (mm^−2^)	.47	.81	.43	.89
	(70)	(46)	(65)	(42)
p53 expression (%)	.18	.29	.43	.11
	(70)	(46)	(65)	(42)
p53 index (mm^−2^)	.73	.99	.90	.90
	(70)	(46)	(65)	(42)
MDR/Pgp	.43	.12	.23	.16
	(70)	(46)	(65)	(42)

^a^Analysis based on disease-specific mortality, and for continuous parameters divided on the median, whereas for MDR/Pgp divided in a chemotherapy-sensitive group (score <2) and a chemotherapy-resistant group (score ≥2). ^b^All kinds of recurrences, but patients with persistent, progressive disease after primary therapy have been deleted from the analyses. ^c^ *P*-values as reported from log-rank tests. ^d^Angiogenesis could not be estimated in 21 cases due to technical, immunohistological problems, see text.
